# The association between symptoms of depression during pregnancy and low birth weight: a prospective study

**DOI:** 10.1186/s12884-020-2842-1

**Published:** 2020-03-06

**Authors:** Xiuxiu Li, Rui Gao, Xiaowei Dai, Hong Liu, Jinxin Zhang, Xuemei Liu, Dongmei Si, Te Deng, Wei Xia

**Affiliations:** 1 Nanshan Maternity & Child Healthcare Hospital of Shenzhen, Shenzhen, China; 2 Sun Yat-Sen University, Guangzhou, China

**Keywords:** Antenatal depression, Low birth weight, Preterm birth, Small for gestational age

## Abstract

**Background:**

Most studies have showed that maternal depression is associated with pregnancy complications. However, there were limited evidences in Chinese population. We examined the associations of antenatal depression symptoms with pregnancy outcomes, especially for low birth weight.

**Methods:**

A total of 1377 singleton pregnant women were recruited from Nanshan Maternity & Child Healthcare Hospital of Shenzhen in this prospective cohort study. Depression symptoms were assessed by the Edinburgh postnatal depression scale (EPDS) questionnaire in the second trimester of gestation; cut-points for the indication of antenatal depression were ≧12 scores in this study. Socio-demographic data, life-style and pregnancy outcomes were collected through Shenzhen Maternity & Child Healthcare database. The risks of adverse outcomes in pregnant women with antenatal depression were determined by multivariate logistic regression and represented as odds ratio(OR) and 95% confidence interval (CI).

**Results:**

Of the 1377 subjects, the prevalence of antenatal depression was 19.1%. The EPDS scores were 13.8 ± 2.0 and 6.5 ± 2.9 (*P* < 0.001) in subjects with and without antenatal depression, respectively. After adjustment for maternal age, education, parity, pre-pregnancy body mass index (BMI), residential area, fetal gender, an EPDS score ≥ 12 (versus. < 12) was associated with an increased risk for low birth weight (odds ratio: 2.05, 95% CI: 1.12–4.64), but not for preterm birth, large for gestational age, small for gestational age or macrosomia.

**Conclusion:**

Pregnant women presenting antenatal depressive symptoms are at elevated risk of low birth weight. Mental health problems of pregnancy should be addressed for the prevention of low birth weight.

## Background

According to the World Health Organization (WHO) [1], depression is one of the main factors for the increase of women disability adjusted life years worldwide. Depression during pregnancy is quite common, with up to 30% of pregnant women experiencing significant depressive symptoms [2, 3]. A systematic review of 28 observational studies (including cross-sectional, cohort, and case-control studies) have estimated that the prevalence of major depression in pregnancy was 3.1–4.9% and that of mild depression was 11% in high-income countries [4]. In low and middle-income countries, the incidence rate of either major or mild depression is more severe [5]. The prevalence of depression in pregnancy reported in China varied between 3.8 and 35.9% due to different screening tools [6–8]. However, diagnosis of depression in pregnant women might be underestimated due to the antenatal hormone levels, and low willingness to participate in such studies for those pregnant women with depression [6]. Therefore, more research on the effective screening or diagnosis of antenatal depression in Chinese pregnant women are warranted.

Depression associated with pregnancy not only damages the physical and mental health of the pregnant woman, but also has an important impact on the future psychological and intellectual development of the fetus [7, 8]. A number of studies have evaluated the impact of depressive symptoms on low birth weight (LBW), which is associated with increased risk of infant mortality and morbidity [9]. However, the results were inconsistent and varied by sample size and the methods used to measure depression [10]. Nonetheless, most of these studies were conducted in high-income countries. Considering the high-speed development of economy and lifestyle transition in China and the potential for ethnic variations, it is important to determine whether antenatal depression is associated with the risk of LBW in the Chinese population. Shenzhen is the city with largest floating population in China, in which pregnant women may experience more pressure, and thus bring more serious mental health problems. This study aims to investigate whether the antenatal depression symptoms is a risk factor for adverse birth outcomes in a Chinese population.

## Methods

### Participants

Participants were recruited from the antenatal clinic in the Shenzhen Nanshan Maternity & Child Healthcare Hospital in China between October 2015 and April 2017. Exclusion criteria were serious disease before pregnancy (eg, hypertensive disorders, diabetes) or obstetric complications (eg, incompetent cervix, fetal abnormality, non-singleton pregnancies). A total of 1500 subjects were enrolled in the present study. Then data on socio-demographic information were collected by interview, and depression symptoms were assessed by the Edinburgh postnatal depression scale (EPDS) questionnaire, in the second trimester. Data on birth outcomes were obtained from the database. Some participants without delivery information on database were contacted via telephone after delivery to obtain information pertaining to birth outcome. Finally, a total of 1377 women were included, and 123 subjects were excluded due to they were not available to be reached via telephone. This study was approved by the ethics committee of the Maternal & Child Healthcare Hospital of Nanshan district in Shenzhen (NO. 2015NSFYEC010), and all participants provided written consent.

### Data collection

#### Demographic characteristics and obstetric conditions

Information on demographic data such as age, occupation and type of household registration were collected by questionnaires. Information on maternal age, pre-pregnancy body mass index (calculated from reported height and weight), number of existing children, high risk pregnancy, smoking history (ever/never),passive smoking and data on alcohol use during pregnancy (yes/no) was recorded at first antenatal care visit. Maternal education was categorized into middle school, high school, college or University and post-graduated or graduate. High-risk pregnancy is defined as pregnancy in which the mother and/or fetus has higher mortality risk than generally healthy counterpart. Causes of high-risk pregnancy included inadequate prenatal care, previous obstetrical history (abortion and spontaneous), pre-existing maternal disease, pregnancy-induced disease (gestational hypertension), and advanced maternal age above 35 years [11].

#### Exposure of depression

Antenatal depression was evaluated during the second trimester using the Edinburgh Postnatal Depression Scale (EPDS) [12, 13]. The EPDS is a 10-item self-report questionnaire to measure the last 7 days of emotional expression. Each question has four options, with a range of 0–3 points. The maximum score is 30 and higher scores suggest more serious depressive symptoms. Although this scale was initially developed for use in a postnatal female population to measure the mood of women after delivery, the effectiveness in antenatal women has been verified by previous studies with excellent reliability [12, 14]. Furthermore, a Chinese translation of the EPDS has been demonstrated to have good reliability and validity, with an internal consistency (Cronbach’s ɑ) of 0.87 [15]. In this study, a cut-off score of 12 was used to identify women with depression [16].

#### Outcomes

Information on pregnancy outcomes was collected at Shenzhen Maternity & Child Healthcare database, which includes antenatal and obstetric data from all pregnant women in Shenzhen city. Outcomes of interest were low birth weight, preterm birth, and small for gestational age. Birth data were obtained from hospital records. Low birth weight (LBW) was defined as birth weight less than 2500 g, and preterm birth (PTB) was less than 37 weeks’ gestation, small for gestational age (SGA) was that birth weight was lower than the 10th percentile of the average weight of the same gestational age [17].

### Statistical analyses

Data were analyzed with SPSS 20.0 for Windows (SPSS Inc., Chicago, IL, USA), and two-sided *p* values of less than 0.05 were considered to be statistically significant. Data were presented as means (and SD) for continuous variables or proportions (%) for categorical variables. Participants were divided into two groups based on EPDS score: a non-depressed group (EPDS score < 12), and a depressed group (EPDS score ≥ 12). The participants’ characteristics were compared by chi-square test or Student *t* test among two groups. The association between depression symptoms and birth outcomes were obtained by chi-square test.

Multivariate logistic regression models were applied to estimate the odds ratios (95% confidence interval) for the risk of low birth weight by comparing the EPDS score≧12 and <12. Adjusted covariates were age, pre-pregnancy BMI, neonatal gender, degree of education, high-risk women and parity. Model1 was not adjusted by previous confounders. Model2 was adjusted for potential confounding variables as above. The variables were entered into the models. A stratified analysis was performed to examine the association of depression symptoms and LBW at different levels of maternal age, residence type, pre-pregnancy BMI and newborn gender.

## Results

### Demographic characteristics information

We approached 1500 women to participate in the study. Questionnaires were all received while 123 women did not give birth in Shenzhen and we were not able to contact them. Therefore, this study reports on a sample of 1377 women (92% of the enrolled sample) who had complete data available for analysis.

In our study, 256 women (19.3%) had antenatal depression. Demographic characteristics of women with and without antenatal depression were compared (Table 1).

**Table 1:** Demographic characteristics of women with and without antenatal depression

	Not depressed (EPDS< 12)		Depressed (EPDS≥12)		*P*
	Mean ± SD or %	Number	Mean ± SD or %	Number	
EPDS score	6.5 ± 2.9	1109	13.8 ± 2.0	265	< 0.001
Age, years					
< 25	4.3	48	7.8	21	0.030
25–34	78.6	872	78.7	211	
≥ 35	17.0	189	13.4	36	
Education					
Under and middle school	3.2	36	3.7	10	0.901
high school	49.7	551	48.9	131	
College or University	42.1	467	43.3	116	
Post-graduated and higher	5.0	55	4.1	11	
Household registration type					
Shenzhen residence	39.0	432	30.6	82	0.032
Temporary residence	58.4	648	67.2	180	
Floating household	2.6	29	2.2	6	
Pre-pregnancy BMI,(kg/m^2^)					
< 18.5	17.9	195	24.9	65	0.076
18.5–23.9	69.5	756	63.2	165	
24–27.9	10.9	118	10.7	28	
> 28	1.7	18	1.1	3	
Passive smoking, Yes	26.0	288	28.0	75	0.773
Parity					
Primigravida	64.7	718	67.2	180	0.455
Multigravida	35.3	391	32.8	88	
High risk pregnancy, Yes	49.1	544	52.2	140	0.349

Women less than 25 years, were more likely to have antenatal depression than their counterparts (*P* < 0.05). A significant difference was found in the type of household residence among the two groups (*P* < 0.05). That is, Antenatal depression symptoms were more common among temporary residents than permanent residents (67.2% vs. 58.4%, *P* < 0.05). However, other variables were not significantly different among the two groups.

### Neonatal outcomes

We found that pregnant women with symptoms of depression had higher rate of LBW than that of non-depressed women in Table 2(*p < 0.05*). However, other adverse birth outcomes did not differ among the two groups.

**Table 2:** Neonatal outcomes of women with and without antenatal depression

	Not depressed (EPDS< 12)		Depressed (EPDS≥12)		*P*
	%	Number	%	Number	
Newborn gender, male	51.8	574	57.8	155	0.074
Preterm birth	4.2	47	6.7	18	0.086
Low birth weight	2.0	22	4.5	12	0.018
Macrosomia^a^	5.2	58	5.6	15	0.810
Small for gestational age^b^	5.2	58	3.4	9	0.201
Large for gestational age^c^	4.2	47	4.9	13	0.659

^a^Macrosomia was that birth weight was heavier than 4000 g

^b^Small for gestational age (SGA) was that birth weight was lower than the 10th percentile of the average weight of the same gestational age

^c^Large for gestational age (SGA) was that birth weight was greater than the 10th percentile of the average weight of the same gestational age

### Depression and risk factors of low birth weight

Univariate Logistic regression analysis was used to evaluate the association between LBW and antenatal depression symptoms in model 1. As shown in Table 3, offspring born women with antenatal depression were more likely to be low birth weight than offspring born to women without antenatal depression (odd ratio [OR]2.39,95% confidence interval [CI]:1.17–4.89). In the model2, after adjusting potential confounders (maternal age, household registration type, high-risk women, passive smoking, primigravida, pre-pregnancy body mass index and male sex), this association was still significant (OR2.05, 95% CI: 1.12–4.6). Stratified analysis showed a significant association when maternal age < 25 and temporary residence (Table 4).

**Table 3:** Odds ratios and 95% CI for the risk of LBW by antenatal depression symptoms

	NBW		LBW	
	Model ^1^	Model ^2^	Model ^1^	Model ^2^
EPDS	1.00	1.00	2.39 (1.17,4.89)	2.05 (1.12,4.64)

^1^Model1 was not adjusted

^2^Model2 was adjusted for pre-pregnancy BMI, neonate gender, maternal age, degree of education, high-risk women and parity

**Table 4:** Odds ratios (95%CI) for depression symptoms on low birth weight stratified by maternal age, residence type, pre-pregnancy BMI and newborn gender

	Model 1	Model 2
	OR (95% CI)	OR (95% CI)
Maternal age^a^		
< 25	4.9 (0.4–57.8)	14.5 (0.6–327.8)
≥ 25	2.1 (0.9–4.4)	2.0 (0.9–4.4)
Residence type^b^		
Shenzhen residence	2.1 (0.5–8.0)	2.2 (0.6–8.7)
Temporary residence	2.3 (1.0–5.4)	2.4 (1.1–5.6)
Pre-pregnancy BMI^c^		
< 25	1.9 (0.9–4.3)	1.8 (0.8–4.3)
≥ 25	6.8 (1.2–37.4)	2.8 (0.1–70.0)
Newborn gender^d^		
Boy	3.6 (1.4–9.0)	2.0 (0.7–6.1)
Girl	1.1 (0.3–4.1)	1.3 (0.3–5.7)

^a^Model 2 was not adjusted maternal age and adjusted for pre-pregnancy BMI, neonate gender, degree of education, high-risk women and parity

^b^Model 2 was not adjusted residence type and adjusted for pre-pregnancy BMI, neonate gender, maternal age, degree of education, high-risk women and parity

^c^Model 2 was not adjusted pre-pregnancy BMI and adjusted for neonate gender, maternal age,degree of education, high-risk women and parity

^d^Model 2 was not adjusted newborn gender and adjusted for pre-pregnancy BMI, maternal age, degree of education, high-risk women and parity

## Discussion

In the present study, an association was observed between antenatal depression symptoms and low birth weight. Women with significantly higher EPDS score were more likely to have low birth weight infants. This association persisted after adjusting for confounders, including pre-pregnancy BMI, neonate gender, maternal age, degree of education, high-risk women and parity. Findings in previous studies regarding the association between antenatal depression and low birth weight remain inconclusive. This disparity may result in different sample size, confounders adjusted, and depression symptoms measurements.

In order to facilitate large-scale epidemiological investigation, EPDS was used as the depression symptom measurement. Although clinician administered diagnostic instruments are the gold standard, they are time-consuming and staff intensive to administer [18]. Conversely, patient-rated screening instruments are easier to use. Because adverse obstetrical outcomes can have significant, long-term, negative health impacts, it would be useful to screen depression at the initial obstetric visit predicts the risk of adverse pregnancy outcomes. EPDS is the only instrument designed to exclude somatic depressive symptoms that are also common symptoms of pregnancy. The effectiveness in antenatal women has been verified by previous studies with excellent reliability [12, 14]. Additionally, a Chinese translation of the EPDS has been demonstrated to have good reliability and validity [15]. We focused on depression symptoms rather than clinically diagnosed depression. The results showed that depression symptoms during pregnancy still had a positive effect on LBW after adjusting several confounders.

Our findings are in keeping with those studies in America black women with large sample size. Some studies [19–21] agreed that depression symptoms are one of the causes of LBW, especially in Neggers’s study [19], depression symptoms rather than depression was concerned. Consistent with our results, *Negger* et al found that women who are depressed during pregnancy are at increased risk of giving birth to low birth weight (AOR =1.4, 95% CI: 1.1–1.7), in which the measure of depression was conducted by the Center for Epidemiologic Studies Depression Scale (CES-D). Although the depression measure was not the same, we both concerned about the depression symptoms rather than the diagnosis of depressive disorder. However, our attention is paid on the second trimester, suggesting that the depressive stage should be concerned and controlled in earlier pregnancy. Additionally, EPDS is more convenient to use in large population survey. Evans’s study used EPDS to screen depression symptoms at 18 and 32 weeks respectively in 13,194 British women, showed that depression symptoms at 18 weeks increased the risk of LBW, but the risk was disappeared after adjusting smoking [22]. It was possible that smoking lies on the causal pathway between depression and intrauterine growth restriction. Adjustment for smoking removed any evidence of a detect association between birth weight and mood during pregnancy. This study mainly focused on white women. We have noticed that the results in different races are conflicting. In a prospective cohort study of 819 African-American women, El-Mohands et al. [20]. Found that women with depression, assessed at < 29 weeks gestational age, had 71% increased odds of LBW in adjusted analysis (OR = 1.71 [95%CI = 1.12,2.62]). Another study among 583 women in Bangladesh [23], showed that depression in the 3rd trimester, assessed via the EPDS, doubled the odds of LBW (OR = 2.24 [95%CI-1.37, 3.68]) in adjusted analysis. Whether the association between depression and LBW are affected by race, socioeconomic level remains to be further explored.

Relevant studies from China and with large sample size are limited. Yang’s case-control study in Wuhan found that antenatal depression or anxiety was not significantly associated with LBW, but individuals with antenatal depression combined with anxiety were at a higher risk of LBW [24]. However, the self-designed questionnaires, rather than a typical screening scale were used in the study for measuring depressive states. Existing reviews tend to suggest that depression, especially in early pregnancy, may be a risk factor for LBW [25, 26]. Our study supported this view, but more evidence is needed.

We found that young mothers (< 25 years of age) seem to be more susceptible to antenatal depression. This might be related to depressive younger pregnant women lacking acknowledgement on pregnant health care. Some studies showed that depression has the tendency to occur in younger pregnant women [27]. We also found that antenatal depression symptoms were more common among temporary residents than permanent residents. This may be related to immigration factors, such as dialect, social isolation, life adaption, unfair opportunities, economic problems or the realization of health care [28].

There are several mechanisms that have been proposed to underlie the association between antenatal depression and neonatal outcomes. A hypothesis has been most extensively investigated which proposed that the hypothalamic-pituitary- adrenocortical axis, which stimulates the release of stress hormones such as cortisol and catecholamines, has a mediation on the association [29]. Study also suggested that the association between self-reported depression and gestational age at birth and fetal growth rate was mediated by antenatal maternal cortisol [30]. Additionally, adverse health behaviors were associated with depression, such as depressed women are more likely to smoke and drink during pregnancy than non-depressed women.

There are limitations in this study. Firstly, depression symptoms were assessed at the second-trimester only, so we probably failed to observe the trend of mood changes throughout the pregnancy. Some women whose depression symptoms occurred at the third-trimester might be misclassified into the non-deprssion group, then the adverse effect of antenatal depression on LBW may be underestimated. Furthermore, data on the history of low birth weight delivery were undocumented. Thus there might be some residual confouders were not adjusted.

## Conclusion

Symptoms of depression in pregnancy significantly increased the risk of low birth weight. More researches on antenatal depression and adverse birth outcomes are needed.

## Data Availability

The datasets used and/or analyzed during the current study are available from the corresponding author on reasonable request.

## Abbreviations

BMI: Body Mass Index
CI: Confidence interval
EPDS: Edinburgh postnatal depression scale
LBW: Low birth weight
NBW: Normal birth weight
OR: Odd ratio
PTB: Preterm birth
SGA: Small for gestational age
